# Press Play to Feel: The Role of Attachment Styles and Alexithymic Features in Problematic Gaming

**DOI:** 10.3390/ijerph20206910

**Published:** 2023-10-11

**Authors:** Andrea Scalone, Gianluca Santoro, Josephin Cavallo, Alessandra Melita, Alessio Gori, Adriano Schimmenti

**Affiliations:** 1 Faculty of Human and Social Sciences, UKE—Kore University of Enna, Piazza dell’Università, 94100 Enna, Italy; andrea.scalone@unikorestudent.it (A.S.); josephin.cavallo@unikorestudent.it (J.C.); alessandra.melita@unikorestudent.it (A.M.); adriano.schimmenti@unikore.it (A.S.); 2 Department of Health Sciences, University of Florence, Via di San Salvi 12, Pad. 26, 50135 Florence, Italy; alessio.gori@unifi.it; 3 Integrative Psychodynamic Psychotherapy Institute (IPPI), Via Ricasoli 32, 50122 Florence, Italy

**Keywords:** problematic gaming, attachment styles, alexithymia, moderation model

## Abstract

Problematic gaming has been consistently associated with insecure attachment styles and alexithymia. However, there is limited knowledge regarding the impact of specific alexithymic features and insecure attachment styles on problematic gaming. The study included a sample of 358 online game players (242 males, 67.6%) between the ages of 18 and 59 (M = 28.46; SD = 8.76) who were recruited from online gaming communities. The participants completed a sociodemographic schedule and measures on attachment styles, alexithymia, and problematic gaming. The results provide evidence for a positive prediction of problematic gaming by dismissing attachment style and the alexithymic factors concerning the difficulty identifying feelings and externally oriented thinking, even when controlling for potentially confounding factors, such as age, sex, education, marital status, and self-reported time devoted to online games. Additionally, the analysis revealed a significant interaction effect between externally oriented thinking and dismissing attachment style in the prediction of problematic gaming. The interaction implies that the alexithymia factors pertaining to externally oriented thinking hold significant relevance in predicting problematic gaming behaviors, especially in cases where dismissive attachment levels are moderately to highly present. These findings emphasize the significance of considering specific insecure attachment styles and alexithymic features when studying problematic gaming behaviors.

## 1. Introduction

Online gaming is a common entertainment activity, with an estimated 2.52 billion gamers as of 2022 [1]. Despite online gaming being usually associated with increased levels of well-being [2], the excessive use of online games might impair the individual’s functioning [3]. Recently, nosographic systems have included clinical syndromes characterized by uncontrolled and dysfunctional online gaming. The American Psychiatric Association [4] included “Internet Gaming Disorder” (IGD) among mental disorders requiring further research in the third section of the Diagnostic and Statistical Manual of Mental Disorders Fifth Edition—Text Revision (DSM-5-TR). The diagnostic criteria for IGD comprise preoccupation, withdrawal, tolerance, failed attempts to control gaming activities, loss of interest in non-gaming activities, persistent engagement in gaming despite psychosocial difficulties, lies about time spent gaming, escapism from negative feelings, and functional impairments. The World Health Organization (WHO) [5] included “Gaming Disorder” (GD) among disorders due to addictive behaviors in the eleventh revision of the International Classification of Diseases (ICD-11). The criteria for GD comprise a diminished interest in daily activities, persistent gaming activities despite negative consequences, a persistent pattern of problematic gaming, exclusion of other mental disorders and/or substance or medication use that might better explain problematic gaming, and functional impairment.

There is some consensus among international experts that GD criteria are more useful for identifying the addictive use of online video games than IGD criteria, which might lead clinicians to overpathologize intensive but non-problematic patterns of gaming [6,7,8]. However, some experts in the field prefer a conceptualization of excessive or otherwise problematic gaming as a maladaptive coping strategy that serves to deal with psychosocial difficulties or distress [9,10]. This latter conceptualization might also serve to avoid classifying transient patterns of excessive gaming as a behavioral addiction [11]. In light of the ongoing theoretical debate pertaining to this subject, the current study adopts the locution “problematic gaming” to encompass self-reported symptoms related to gaming activities, regardless of the existing nosographic classification. This decision is based on the opportunity to investigate, within a non-clinical setting, the particular set of symptoms that are believed to be linked to problematic videogame use, considering potential issues related to gaming from a dimensional standpoint.

Research findings have demonstrated a positive association between problematic gaming and an increased amount of time spent online [12,13], as well as various psychological difficulties. Among other manifestations, people who display problematic gaming tend to exhibit a diminished self-esteem [14,15,16], maladaptive personality traits [17,18], emotion dysregulation [19,20,21], and maladaptive metacognitive beliefs [22].

Notably, an increasing body of scientific literature indicates that insecure attachment styles are also implicated in various problematic online behaviors [23,24,25], including problematic gaming [26,27]. Attachment is a motivational system that leads individuals to seek proximity to caregivers and close others in order to obtain safety from dangers and relief from distress [28,29]. Early interactions between children and their caregiver shape the child’s representations of the self, others, and the relationships between the self and others—also termed “internal working models” (IWMs [30]). Bartholomew and Horowitz [31] identified four adult attachment styles based on the representations of the self and others: secure, dismissing, preoccupied, and fearful. Individuals with a secure attachment style perceive themselves and others positively, and feel comfortable within close relationships. Individuals with a dismissing attachment style perceive themselves positively but others negatively, and tend to avoid intimacy. Individuals with a preoccupied attachment style perceive themselves negatively but others positively, which leads to experiencing high levels of anxiety in close relationships. Finally, individuals with a fearful attachment style perceive both themselves and others negatively, displaying high levels of both anxiety and avoidance in close relationships.

The research indicates that a secure attachment style may serve as a protective factor against problematic gaming. Indeed, previous studies have demonstrated significant and negative associations between the quality of attachments to the mother or peers and the levels of problematic gaming [32,33]. Additionally, while some research suggests that individuals with an anxious attachment attitude, particularly a preoccupied attachment style, may be more prone to developing problematic gaming [26,34,35], there is evidence to suggest that avoidant attitudes within intimate relationships may also contribute to the problem [27,36]. For example, Santoro and colleagues [34] examined the relationships between parental bonding, attachment styles, and problematic gaming in a sample of adult videogame players: they found that a preoccupied attachment style was a full mediator of the positive relationship between maternal overprotection and problematic gaming. Sung and colleagues [27] found that both avoidant and anxious attachment attitudes predicted higher levels of problematic gaming through the mediating effects of stressful events in a sample of university students. Monacis and colleagues [36] conducted a study examining the influence of different characteristics associated with anxious and avoidant attachment attitudes on problematic gaming among adolescents and young adults: their findings revealed that individuals who exhibit a secure attachment style, characterized by a feeling of confidence in close relationships, and those who experience discomfort with intimacy, linked to an avoidant attachment attitude, were more likely to have lower levels of problematic gaming; conversely, individuals with an excessive need for approval (an anxious attachment attitude) and those who perceive relationships as secondary (an avoidant attachment attitude) were more likely to engage in problematic gaming. Interestingly, the preoccupation with relationships did not have a significant impact on problematic gaming in that study.

Alexithymia has also been linked to problematic gaming [37,38]. Alexithymia is a personality trait characterized by difficulties identifying and describing feelings, as well as a tendency towards externally oriented thinking [39]. There is evidence that alexithymia may increase the risk of engaging in addictive behaviors, likely as a means to cope with unprocessed and dysregulated feelings [40,41,42,43,44]. Accordingly, previous research has highlighted a significant and positive association between alexithymia and increased problematic gaming [45,46,47]. However, little is known about the role played by specific alexithymic features (namely, difficulty identifying feelings, difficulty describing feelings, and externally oriented thinking) in the prediction of problematic gaming. In fact, studies investigating alexythimia in patients suffering from gaming disorder suggests that about a third of these individuals display clinically relevant levels of alexithymia (34.2% in the study by Pape and colleagues [38]) and increased scores in each alexithymia factors. However, when the effect of specific alexithymic features on problematic gaming are examined in research, the findings are more difficult to interpret. For example, Bonnaire and Baptista [37] found that problematic gamers tend to display higher levels of alexithymic features when compared to individuals without gaming problems. Additionally, their research indicated that only alexithymia itself, and not the underlying features of alexithymia, predicted the presence of clinically significant levels of problematic gaming. In contrast, Maganuco and her colleagues [45] found that increased difficulties identifying and describing feelings were significant predictors of increased problematic Internet use among MMORPG (massively multiplayer online role-playing game) players.

In line with previous research, we hypothesized that:

(1) Higher scores in the secure attachment style would be associated with lower scores in problematic gaming;
(2) Higher scores in insecure attachment styles would be associated with higher scores in problematic gaming;
(3) Higher scores in alexithymic features would be associated with higher scores in problematic gaming.

Furthermore, we aimed to explore the interaction between alexithymic features and adult attachment styles in the prediction of problematic gaming symptoms. Earlier studies suggest that individuals who exhibit high scores in insecure attachment styles and heightened alexithymic traits may be more susceptible to engaging in problematic gaming, perhaps as a way to alleviate interpersonal challenges and difficulties in managing emotions. However, there is a gap in knowledge regarding the specific attachment styles and alexithymic features that might contribute to the development of problematic gaming, as well as how these dimensions interact to produce negative outcomes in terms of problematic gaming symptoms.

Finally, building upon previous research that has established notable links between sociodemographic factors (e.g., gender, age, and marital status) and the occurrence of problematic online behaviors [48,49,50], with particular emphasis on the increased susceptibility of younger, unmarried males to exhibit signs of problematic gaming [35,51], our study additionally seeks to elucidate the association between insecure attachment styles, alexithymic features, and problematic gaming, while accounting for time spent gaming and these sociodemographic variables in the analysis.

## 2. Materials and Methods

### 2.1. Participants and Procedure

The study sample comprised 358 online game players (242 males, 67.6%). The participants were between 18 and 59 years old (M = 28.46, SD = 8.76), and 144 (40.2%) of them were married. The average of the years of education was 13.77 (SD = 2.55). The participants reported spending an average of 2.92 h a day (SD = 1.89) playing online games. Among the participants, 243 individuals (67.9%) used multiplayer online game services, 199 individuals (55.6%) were engaged in massively multiplayer online (MMO) games, 126 individuals (35.2%) were engaged in online casual games, 73 individuals (20.4%) were engaged in browser games, and 32 individuals (8.9%) were engaged in multi-user dungeon (MUD) games. Females reported more years of education than males (*t*_(356)_ = −2.04, *p* = 0.04). Additionally, a higher prevalence of married participants was observed among females (*χ^2^*_(1)_ = 5.67, *p* = 0.02). No significant sex difference was found for age (*t*_(356)_ = −0.36, *p* = 0.72) and time spent gaming (*t*_(356)_ = 1.62, *p* = 0.11). The recruitment of the participants was conducted from 1 June 2022 to 30 January 2023. The participants were recruited through advertisements posted on 146 Facebook communities for Italian online game players. Facebook communities were primarily centered on multiplayer online game services (*n* = 114), MMO games (*n* = 19), non-specific online games (*n* = 8), casual games (*n* = 3), and browser games (*n* = 2). All advertisements were linked to an anonymous online survey that included an informed consent, a sociodemographic schedule, and self-report measures on problematic gaming, attachment styles, and alexithymia. People who signed the electronic informed consent were automatically administered the sociodemographic schedule and self-report measures. No compensation was provided. The study received ethical clearance and was conducted in accordance with the Declaration of Helsinki.

### 2.2. Measures

The *Internet Gaming Disorder Scale-Short Form* (IGDS9-SF [52,53]) is a self-report measure that assesses problematic gaming in accordance with the IGD diagnostic criteria. It includes 9 item rated on a 5-point Likert scale (1 = “never”, 5 = “very often”). An example of an item is: “Have you continued your gaming activity despite knowing it was causing problems between you and other people?”. The IGDS9-SF has demonstrated adequate psychometric properties in several countries [54]. In the current study, the Cronbach’s alpha coefficient of the IGDS9-SF was 0.83.

The *Relationship Questionnaire* (RQ [31,55]) is a self-report measure that assesses adult attachment styles, that is, secure, dismissing, preoccupied, and fearful. Participants are asked to indicate their agreement with four first-person statements by rating a 7-point Likert scale (1 = “strongly disagree”, 7 = “strongly agree”). An example of an item is: “I am comfortable without close emotional relationships. It is very important to me to feel independent and self-sufficient, and I prefer not to depend on others or have others depend on me” (related to dismissing attachment style). The RQ has demonstrated a good discriminant validity [56] and test–retest reliability [57].

The *Toronto Alexithymia Scale-20 items* (TAS-20 [58,59,60]) is a self-report measure that assesses alexithymic features, that is, difficulty identifying feelings (DIF), difficulty describing feelings (DDF), and externally oriented thinking (EOT). It consists of 20 items rated on a 5-point Likert scale (1 = “strongly disagree”, 5 = “strongly agree”). An example of an item is: “I am often confused about what emotion I am feeling” (related to DIF). The TAS-20 is the most commonly used measure for evaluating alexithymia, and consistent evidence supported its psychometric properties [61]. In the current study, the Cronbach’s alpha coefficient was 0.83 for the TAS-20 total scale, 0.87 for the DIF scale, 0.76 for the DDF scale, and 0.54 for the EOT scale.

A sociodemographic schedule was employed to collect information on sex, age, years of education, marital status, time spent gaming, and videogame-related activities.

### 2.3. Statistical Analyses

After conducting the literature review, we determined a conservative estimate for the expected correlation coefficients among the variables of interests as *r* = 0.20, which was used to calculate the sample size. By setting the Type I and Type II error rates at 0.05, it was determined that a sample of *N* = 319 was the minimal sample size for the study. Following data collection, descriptive statistics were computed for all the study variables. Parametric analyses were then employed due to the indication of normally distributed data (as reflected by absolute skewness and kurtosis values that were below the rule-of-thumb cutoffs typically applied to sample sizes larger than 300 participants [62]). Sex differences for problematic gaming were examined through a *t*-test. Associations between problematic gaming and the other variables of interest were investigated through Person’s *r* correlations. A multiple linear regression analysis was performed to examine the effects of the sociodemographic characteristics (i.e., sex, age, years of education, and marital status), self-reported time spent gaming, attachment styles, and alexithymic features on problematic gaming. Finally, moderation analyses were performed to test whether interactions between alexithymic features and attachment styles predicted problematic gaming. A variable can be identified as a moderator if it influences the size, direction, or strength of the effect that another variable has on a different variable. Therefore, a moderation analysis examines the effects resulting from the interaction between two variables on another variable; consequently, conducting a moderation analysis enables the identification of specific conditions that influence the relationship between two variables. Moderation analyses were performed employing Model 1 of the PROCESS Macro for SPSS [63]. The data analytic plan stipulated that the alexithymic traits would be treated as independent variables (taking into account that alexithymia is a trait), and the specific attachment styles determined to be significant predictors in the multiple regression analysis were regarded as moderators in the analysis. Sociodemographic characteristics, time spent gaming, and the other alexithymic features were entered as covariates in all moderation models. The effects of the independent variables on the dependent variable were examined at low (−1 SD), moderate (mean), and high (+1 SD) levels of the moderator variable. Scores on independent variables and moderator were mean-centered in order to avoid multicollinearity [64]. A critical level of statistical significance was established with a *p*-value of 0.05. In the case where the 95% confidence interval incorporates the value of 0, it indicates that the effect is not statistically significant at the 0.05 level. Conversely, if the interval does not contain 0, it suggests that the effect is statistically significant at the 0.05 level.

## 3. Results

The descriptive statistics are shown in Table 1. No significant sex differences were observed for problematic gaming (*t*_(356)_ = −1.42, *p* = 0.16).

Significant associations were found through Pearson’s *r* correlations. Problematic gaming was significantly and positively associated with time spent gaming (*r* = 0.32; *p* < 0.01), preoccupied (*r* = 0.19, *p* < 0.01) and fearful (*r* = 0.19, *p* < 0.01) attachment styles, and alexithymia (*r* = 0.42, *p* < 0.01), including DIF (*r* = 0.39, *p* < 0.01), DDF (*r* = 0.30, *p* < 0.01), and EOT (*r* = 0.26, *p* <0.01). Additionally, problematic gaming was significantly and negatively associated with age (*r* = −0.12, *p* = 0.03) and secure attachment style (*r* = −0.16, *p* < 0.01).

The results of the multiple linear regression analysis are displayed in Table 2. Male sex, self-reported time spent gaming, dismissing attachment, DIF, and EOT were predictively associated with increased levels of problematic gaming.

**Figure 1:**
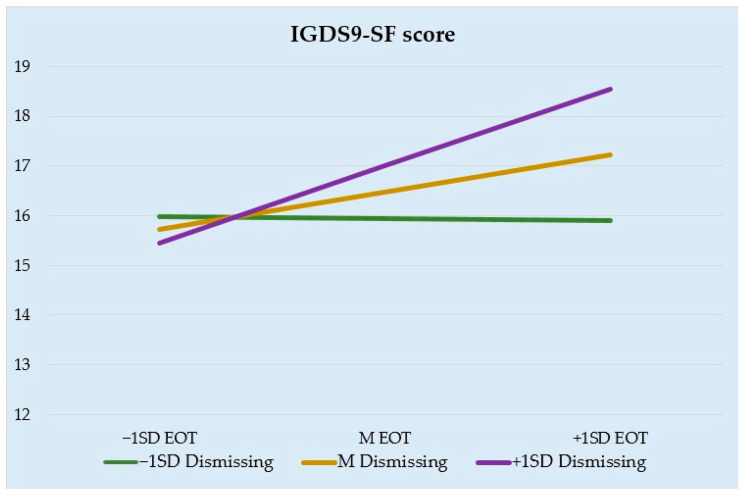
Interaction of externally oriented thinking and dismissing attachment style in predicting problematic gaming scores.

Then, we performed a moderation analysis to examine the interaction between alexithymia total score and dismissing attachment style scores in predicting problematic gaming. This analysis provided evidence of prediction, with a significant interaction between the two variables (B = 0.0243, se = 0.0115, *p* = 0.0363, 95% CI [0.0016, 0.0470]). Subsequently, three moderation analyses were computed to further investigate the specific effects of the alexithymic features and dismissing attachment style on problematic gaming. Thus, we tested whether the interactions between DIF and dismissing attachment style, between DDF and dismissing attachment style, and between EOT and dismissing attachment style predicted problematic gaming. The effects of interactions were controlled for sociodemographic covariates, self-reported time spent gaming, and other alexithymic features. In the first model, DIF (B = 0.3736, se = 0.0546, *p* < 0.0001, 95% CI [0.2662, 0.4810]) positively predicted problematic gaming; however, the effect of dismissing attachment style on problematic gaming was not significant (B = 0.2798, se = 0.1441, *p* = 0.0531, 95% CI [−0.0037, 0.5633]). Additionally, the interaction between DIF and dismissing attachment style was not significant (B = 0.0270, se = 0.0227, *p* = 0.2358, 95% CI [−0.0177, 0.0716]). Male sex (B = −1.4893, se = 0.5984, *p* = 0.0133, 95% CI [−2.6663, −0.3123]), reported time spent gaming (B = 1.0502, se = 0.1468, *p* < 0.0001, 95% CI [0.7615, 1.3390]), and EOT (B = 0.1681, se = 0.0649, *p* = 0.0100, 95% CI [0.0405, 0.2956]) were significant covariates into the model. In the second model, DDF (B = 0.0534, se = 0.0742, *p* = 0.4723, 95% CI [−0.0925, 0.1993]), dismissing attachment style (B = 0.2837, se = 0.1443, *p* = 0.0501, 95% CI [−0.0001, 0. 5676]), and their interaction (B = 0.0370, se = 0.0299, *p* = 0.2171, 95% CI [−0.0218, 0.0958]) were not significant predictors of problematic gaming; in contrast, male sex (B = −1.4675, se = 0.5981, *p* = 0.0146, 95% CI [−2.6437, −0.2912]), reported time spent gaming (B = 1.0392, se = 0.1467, *p* < 0.0001, 95% CI [0.7506, 1.3279]), DIF (B = 0.3752, se = 0.0546, *p* < 0.0001, 95% CI [0.2678, 0.4826]), and EOT (B = 0.1704, se = 0.0648, *p* = 0.0089, 95% CI [0.0429, 0.2978]) were associated with increased levels of problematic gaming. In the third model, EOT (B = 0.1609, se = 0.0642, *p* = 0.0127, 95% CI [0.0346, 0.2873]) was positively associated with problematic gaming, but dismissing attachment style (B = 0.2762, se = 0.1424, *p* = 0.0532, 95% CI [−0.0039, 0.5564]) had no significant direct effects on problematic gaming; however, the interaction between EOT and dismissing attachment style proved significant in positively predicting problematic online gaming (B = 0.0882, se = 0.0303, *p* = 0.0038, 95% CI [0.0286, 0.1478]), especially at moderate (B = 0.1609, se = 0.0642, *p* = 0.0127, 95% CI [0.0346, 0.2873]) and high (B = 0.3305, se = 0.0843, *p* = 0.001, 95% CI [0.1648, 0.4963]) levels of dismissing attachment style. In this model, male sex (B = −1.3229, se = 0.5943, *p* = 0.0266, 95% CI [−2.4918, −0.1541]), reported time spent gaming (B = 1.0132, se = 0.1456, *p* < 0.0001, 95% CI [0.7268, 1.2996]), and DIF (B = 0.3647, se = 0.0541, *p* < 0.0001, 95% CI [0.2582, 0.4712]) were also associated with increased problematic gaming. The slopes representing the predicted scores of problematic gaming at different levels of EOT and dismissing attachment style—specifically, at values equivalent to one standard deviation below the mean (−1SD), values equivalent to the mean (M), and values equivalent to one standard deviation above the mean (+1SD)—are displayed in Figure 1. The analysis of the slope curves indicates that, at varying levels of EOT, low levels of dismissing attachment style are not significantly associated with problematic gaming; in contrast, higher levels of dismissing attachment style, specifically moderate and high levels, are linked to a greater likelihood of increased problematic gaming, contingent on higher levels of EOT.

## 4. Discussion

The objective of this study was to examine the effects of alexithymic features and adult attachment styles, along with their interactions, on problematic gaming behavior in a sample of adult videogame players. The literature suggests that sociodemographic characteristics and time spent gaming might be involved in problematic gaming. Accordingly, we examined the role of these variables in our sample. Despite previous research having showed that males tend to report higher levels of problematic gaming than females [65], no significant sex differences were observed in our sample. This finding offers different potential explanations. The finding could be ascribed to our sample selection process, which only included individuals from gaming communities; as a result, most study participants might be highly involved with gaming, with some of them exhibiting problematic gaming symptoms regardless of their sex. Alternatively, this finding could suggest that males and females in our sample had distinct perceptions and responses to symptoms of problematic gaming, influenced by their individual understanding of the impairments gaming can cause [66]. Consistent with the existing literature, the study revealed a significant association between problematic gaming and younger age [35,67] as well as increased hours spent gaming [13,68,69].

The first hypothesis of the current study was confirmed, as we found a negative association between problematic gaming scores and secure attachment style scores. However, the second hypothesis of the study was only partially supported. In fact, problematic gaming was associated with preoccupied and fearful attachment styles, which are both characterized by high levels of anxiety in close relationships, but the correlation between problematic gaming and dismissing attachment style was not significant. These correlational findings support earlier studies positing that secure attachment may mitigate the risk of problematic gaming [36,70], and suggest that individuals with anxious attitudes in close relationships may be more prone to engaging in excessive gaming [26,34], perhaps as a compensatory strategy to establish social bonds and alleviate distress in close relationships [71,72]. Additionally, the third hypothesis of our study was confirmed, as we observed positive associations between problematic gaming and the examined alexithymic features (i.e., DIF, DDF, and EOT). These findings are aligned with prior research indicating that some individuals may excessively turn to online gaming as a means of evading emotions that they may find difficult to process [45].

Surprisingly, despite correlation analyses displaying no significant association between dismissing attachment style and problematic gaming, when controlling for sociodemographic covariates, time spent online, alexithymic features, and the other attachment styles in multiple regression analysis, dismissing attachment was a significant predictor of problematic gaming. Specifically, we found that male sex, time spent gaming, dismissing attachment, DIF, and EOT predicted increased problematic gaming. Moderation analyses provided some clarification concerning this counterintuitive finding. These analyses showed that the direct effect of dismissing attachment on problematic gaming was not significant; however, dismissing attachment interacted with EOT in predicting higher scores in problematic gaming, especially at moderate to high levels of dismissing attachment. This suggests that individuals with dismissing attachment who also display increased levels of the operatory thinking implied in the EOT factor of alexithymia might be particularly prone to developing symptoms of gaming disorders.

Therefore, our findings provide new insights on the role of avoidant attachment attitudes in problematic gaming. In fact, some research has shown that both anxious and avoidant attachment attitudes are involved in problematic gaming patterns [27,36], whereas other evidence has suggested that only anxious attachment attitudes positively predict problematic gaming [34,35]. Our study has the potential to contribute to a clearer understanding of these contradictory findings. Specifically, our research supports the hypothesis that the alexithymic feature concerning a difficulty identifying and discerning emotions (DIF) might facilitate problematic gaming behaviors [45], but also indicates that increased levels of a dismissing attachment style, characterized by a positive view of the self that is coupled with a negative view of others—thus, an avoidant disposition towards intimate relationships—may interact with an operational, externally directed cognitive style (EOT) to heighten the susceptibility to problematic gaming. Within the framework of a compensatory conceptualization of problematic gaming patterns, it is plausible to consider that some intrinsic characteristics associated with a dismissing attachment style, such as a proclivity for self-reliance and a tendency to avoid intimacy [31], converge with a propensity to focus attention predominantly on practical concerns and external events rather than feelings. Consequently, individuals exhibiting a dismissing attachment may redirect their attention towards virtual environments and employ online gaming as a coping mechanism to curtail their engagement in interpersonal interactions and regulate their negative feelings [73,74].

Finally, male sex and larger amount of time spent gaming were significant covariates in the moderation models, suggesting that the model predictions might be more relevant for the male population of highly engaged gamers. Additionally, previous studies showed that a larger amount of time spent gaming is associated with increased problematic gaming [13,68,69]. It is noteworthy that the amount of time spent gaming may also depend on the extent of the individual’s psychological problems and difficulties—which are represented by dismissing attachment styles, EOT, and DIF in the current study [9,66].

This study has some limitations. The sample consisted of online gamers from the online community. Hence, it is important to note that our findings should not be directly generalized to individuals who have been diagnosed with gaming disorder. Furthermore, the variables of interests were assessed through well-validated measures, but the use of self-report instruments may increase the risk of bias. Therefore, it is recommended that future studies include individuals who have received a diagnosis of gaming disorder and employ clinical interviews in order to mitigate the potential for measurement errors. In addition, the cross-sectional design of the study did not allow us to ascertain the causal relationships between alexithymic features, attachment styles, and problematic gaming. Longitudinal studies are greatly needed to detect the effects of attachment styles and alexithymic traits on problematic gaming. Notably, the current study did not investigate the preference towards specific online games. Previous research suggests that specific psychological vulnerabilities may increase the maladaptive engagement in certain types of online games [75]. Accordingly, future research might examine whether attachment styles and alexithymic features foster the maladaptive use of certain types of online games.

## 5. Conclusions

Its limitations notwithstanding, the current study extends previous research on the role of attachment styles and alexithymia in problematic gaming. Based on our findings, it appears that certain individuals with difficulties identifying their own feelings and an externally oriented thinking are prone to displaying an excessive gaming behavior. This behavior might serve as a compensatory strategy to avoid directly engaging with their emotions. Additionally, increased levels of dismissing attachment style might foster this tendency to divert attention towards virtual environments rather than one’s own internal world, increasing the risk for problematic gaming.

We believe that it is crucial to understand the psychological difficulties underlying problematic gaming, rather than solely focusing on its symptoms, in order to effectively implement tailored interventions. Accordingly, our results may have relevant implications for the assessment and treatment of problematic gaming. It is advisable that clinicians carefully evaluate whether problematic gaming might constitute a compensatory strategy to deal with unprocessed feelings and interpersonal difficulties. In this context, clinical interventions should be aimed at helping patients to recognize and regulate their own feelings and interpersonal difficulties in order to reduce their problematic gaming patterns and enhance their interpersonal abilities. Nevertheless, gamers with a dismissing attachment style, due to their negative view of others, may experience feelings of mistrust towards clinicians. Therefore, it is recommended that clinicians adopt a non-judgmental stance towards these patients, recognizing that their problematic gaming behaviors may stem from underlying psychological challenges. Such an approach could foster a sense of trust in individuals who excessively engage in gaming, ultimately enhancing their adherence to treatment goals.

## Figures and Tables

**Table 1:** Descriptive statistics ^1^.

	Full Sample (*N* = 358)				
	M	(SD)	Range	Skewness	Kurtosis
Age	28.46	(8.76)	18–59	1.26	1.19
Years of education	13.77	(2.60)	8–21	−0.01	0.82
Time spent gaming (hours)	2.92	(1.89)	0–10	1.18	1.70
RQ—Secure attachment style	3.70	(1.97)	1–7	0.18	−1.20
RQ—Dismissing attachment style	4.30	(1.92)	1–7	−0.20	−1.13
RQ—Preoccupied attachment style	3.42	(2.07)	1–7	0.32	−1.29
RQ—Fearful attachment style	3.29	(2.09)	1–7	0.40	−1.22
TAS-20—Alexithymia (total score)	43.41	(11.98)	20–82	0.63	0.08
TAS-20—Difficulty identifying feelings	13.43	(6.15)	7–31	0.91	−0.18
TAS-20—Difficulty describing feelings	12.04	(4.73)	5–25	0.37	−0.79
TAS-20—Externally oriented thinking	17.93	(4.67)	8–32	0.24	−0.34
IGDS9-SF—Problematic gaming	16.56	(6.10)	9–37	1.05	0.82

^1^ RQ = *Relationship Questionnaire*, TAS-20 = *Toronto Alexithymia Scale*, IGDS9-SF = *Internet Gaming Disorder Scale—Short Form*.

**Table 2:** Linear regression model predicting the severity of problematic gaming ^2^.

	β	SE	Partial *r*	*t*	*p*
Sex	−0.10	0.61	−0.12	−2.23	0.03
Age	−0.02	0.04	−0.02	−0.37	0.71
Years of education	−0.01	0.11	−0.01	−0.11	0.91
Marital status	0.04	0.60	0.04	0.82	0.42
Time spent gaming (hours)	0.33	0.15	0.36	7.09	<0.01
RQ—Secure attachment style	−0.02	0.16	−0.02	−0.44	0.66
RQ—Dismissing attachment style	0.11	0.15	0.12	2.22	0.03
RQ—Preoccupied attachment style	0.08	0.15	0.09	1.66	0.10
RQ—Fearful attachment style	−0.04	0.16	−0.04	−0.72	0.47
TAS-20—Difficulty identifying feelings	0.37	0.06	0.32	6.37	<0.01
TAS-20—Difficulty describing feelings	0.04	0.08	0.04	0.66	0.51
TAS-20—Externally oriented thinking	0.13	0.07	0.14	2.60	0.01

^2^ Sex = “male” was coded as 1, “female” was coded as 2, RQ = *Relationship Questionnaire*, TAS-20 = *Toronto Alexithymia Scale*, IGDS9-SF = *Internet Gaming Disorder Scale—Short Form*; Model: F(12,345) = 13.67; *p* < 0.001, R^2^ = 0.32.

## Data Availability

The data presented in this study are available upon request from the first author. The data are not publicly available due to GDPR 2016/79.
